# Interaction among susceptibility genotypes of *PARP1* SNPs in thyroid carcinoma

**DOI:** 10.1371/journal.pone.0199007

**Published:** 2018-09-05

**Authors:** Kashif Bashir, Romana Sarwar, Soma Saeed, Ishrat Mahjabeen, Mahmood Akhtar Kayani

**Affiliations:** Department of Biosciences, COMSATS Institute of Information and Technology, Islamabad, Pakistan; 2nd medical school of Charles University, CZECH REPUBLIC

## Abstract

Polymorphisms in DNA repair genes may alter the repair mechanism which makes the person susceptible to DNA damage. Polymorphic variants in these DNA repair pathway genes such as Poly (ADP-ribose) polymerase- 1 (*PARP1*) have been associated with susceptibility of several types of cancer including thyroid. Many studies have been published on *PARP1* gene polymorphisms and carcinogenesis with inconsistent results. The present study was designed to explore the link between the *PARP1* polymorphisms and thyroid cancer risk. This case-control study was comprised of 456 thyroid cancer patients and 400 healthy controls. Three SNPs of *PARP1* gene; rs1136410, rs1805414 and rs1805404 were analyzed using ARMS-PCR. The combined genotype and haplotype analysis were performed using haploview software 4.2. Major allele homozygote (CC) of rs1136410 and combined genotype (TT+TC) of rs180414 showed a significant association with thyroid cancer risk (OR = 1.30; 95% CI 0.99–1.77; P = 0.05) and (OR = 0.43; 95% CI = 0.27–0.67; P = 0.03). Histological subtype analysis showed the significant association of selected *PARP1* SNPs with papillary, follicular and anaplastic subtypes in thyroid cancer patients. Haplotype analysis showed that TCT (p = 0.01), CTT (p = 0.02) and CTC (p = 0.03) were significantly higher in controls when compared to cases. However, TTC (p = 0.05) and TCC (p = 0.01) haplotype frequency was significantly higher in cases compared to controls. Global haplotype analysis showed that there was an overall significant difference between cases and controls (p = 0.001). Identification of these genetic risk markers may provide evidence for exploring insight into mechanisms of pathogenesis and subsequently aid in developing novel therapeutic strategies for thyroid cancer.

## Introduction

Most frequent malignancy of thyroid gland is thyroid cancer which has most increasing trend in Pakistan and throughout the world [1]. The incidence of thyroid cancer in Pakistan is 1.2% of all malignancies consisting of papillary thyroid carcinoma 69–71%, follicular thyroid carcinoma 11–13%, medullary thyroid carcinoma 3–5% and anaplastic thyroid carcinoma 1–2% [2]. This trend is alarming in Asia and Europe [3], Canada [4], UK [5] and USA [6] despite good survival rate. Overall incidence of thyroid cancer is 1–2% globally [7].

Disruptions in DNA repair pathways predispose cells to DNA damage and accumulation of this damage can cause cancer and may promote the cancer growth [8]. Among the five different DNA repair pathways, base excision repair pathway (BER) is the main pathway involved in repairing of DNA damage [9, 10]. Variations in BER pathway and their mechanisms have been linked with increased risk of different cancers including thyroid cancer. BER pathway includes different molecules including Poly (ADP-ribose) polymerase- 1 (*PARP1*) also known as adenosine diphosphate ribosyl transferase, a significant component of BER system [11]. It is located on chromosome 1q^41–^q^42^, encoding a 113 KDa zinc finger DNA binding protein. Poly (ADP-ribosyl) transferase can modify various nuclear protein by poly (ADP-ribosylation [12]. A total of 1287 SNPs of *PARP1* gene has been reported of which including 202 in coding known as coding region SNPs (cSNPs). In all cSNPs of *PARP1* gene, Val762Ala polymorphism (rs1136410) is the most studied SNP but the cSNPs Ala284Ala (rs1805414) and Asp81Asp (rs1805404) are least investigated. *PARP1* Val762Ale is based on T to C change at codon 762 at exon 17 in which valine is replaced with alanine in catalytic domain of *PARP1* gene. *PARP1* VAL762ALE has been reported to be associated with altered activity of *PARP1* gene [11,13,14]. Change in amino acid results in reduced activity of *PARP1* gene, which ultimately relates to increased risk of different cancers [12– 15]. Few studies have also shown positive association of *PARP1* SNPs (rs180404 and rs180414 at position 81 and 284) with Alzheimer’s disease [16], glioblastoma [17] and protectively associated with colorectal cancer [18, 19]. Nevertheless, inconsistent results of all three SNPs have failed to clarify the complicated relationships between *PARP1* and thyroid cancer risk. Additionally, the *PARP1* Val762Ale has been showed combine effect with various SNPs in carcinogenesis of different region such as gastric region [20], colorectal region [21], breast region [22], brain region [23] and bladder region [24].

To best of our knowledge, limited number of studies have been reported on the association of *PARP1* gene SNPs rs3611410 (Val762Ala), rs1805414 (Ala284Ala) and rs1805404(Asp81Asp) with thyroid cancer in Pakistani population. Therefore, to explore the reliable influence of these SNPs of *PARP1* on thyroid cancer, mutational analysis in thyroid cancer patients and healthy controls has been planned to assess the active involvement of these polymorphisms in carcinogenesis.

## Materials and methods

### Study subjects and

Present study comprised of 456 confirmed patients of thyroid cancer and healthy control individuals of same age and gender (Table 1). Diagnosed thyroid cancer patients were confirmed through histopathological test at Nuclear medicine department of NORI (Nuclear Oncology Radiation Institute) Islamabad and PIMS (Pakistan institute of medicine Sciences). Normal individual who came for routine check-up were sampled as controls. Previously diagnosed patients of any cancer were excluded from this study. Written consent was taken from the individuals and questionnaire (S1 Annex) filled with data related to demographic factors, addictions, and eating habits was collected. Specimens of peripheral blood from all individuals were collected.

**Table 1:** Demographic characteristics for controls and cases.

Variables	Case (n = 456)	Controls (n = 400)	P*-value
Age (Y)			
<42, n(%)	208 (45.6)	179 (44.75)	0.87
>42, n(%)	248 (54.38)	221 (55.25)	0.89
Sex			
Male, n(%)	107 (23.4)	70 (17.5)	0.08
Female, n(%)	349 (76.53)	330 (82.2)	0.46
Grade of cancer			
Grade I	211 (46%)	N/A	0.08
Grade II	162 (36%)	N/A	
Grade III	72 (15%)	N/A	
Grade IV	11 (3%)	N/A	
Family History			
Yes, n(%)	32 (7.2)	6 (1.5)	0.0004
No, n(%)	427 (93.7)	394 (98.5)	0.0004

n = total number; P* = χ2-test.

### Ethical approval

The study was conducted with a prior approval from the institutional ethical review board of COMSTAS Institute of Information Technology (CIIT) Islamabad. Members of this committee include Dean ORIC (Office of Research Innovation and Commercialization) Prof. Dr. Raheel Qamar (convener), Prof. Dr. Mahmood A Kayani (Chairman Deptt of Biosciences), Dr. Faheem Tahir (Deputy Director, NIH) and Dr. Tayyaba Yasmin (Associate Head of department). All the samples were collected after a signed, informed consent from all participants of the study. The ethical review board approved the consent procedure &execution of project on thyroid cancer.

### DNA extraction

About 3-4ml blood from all individuals were taken. Extraction of DNA from whole blood was performed according to Phenol chloroform method with minor modifications [25]. Quantification of extracted DNA was done through 2% ethidium bromide gels and spectrophotometrically using Nano Drop (Thermoscientifiv, USA). DNA samples were stored at -20°C.

### SNPs selection

Three functional polymorphisms of *PARP1* gene were selected using a set of web-based SNP selection tools (http://snpinfo.niehs.nih.gov/snpinfo/snpfunc.htm). Following criteria was followed for selection of functional SNPs: (1) Minor allele frequency of validated SNPs > 5% in Asian population; (2) validated SNPs in important functional domain of *PARP1* gene such as Val762Ala (rs3611410, catalytic domain), Ala284Ala (rs1805414, PARD1 domain) and Asp81Asp (rs1805404, Zinc figure domain).

### Genotyping

**Fig 1:**
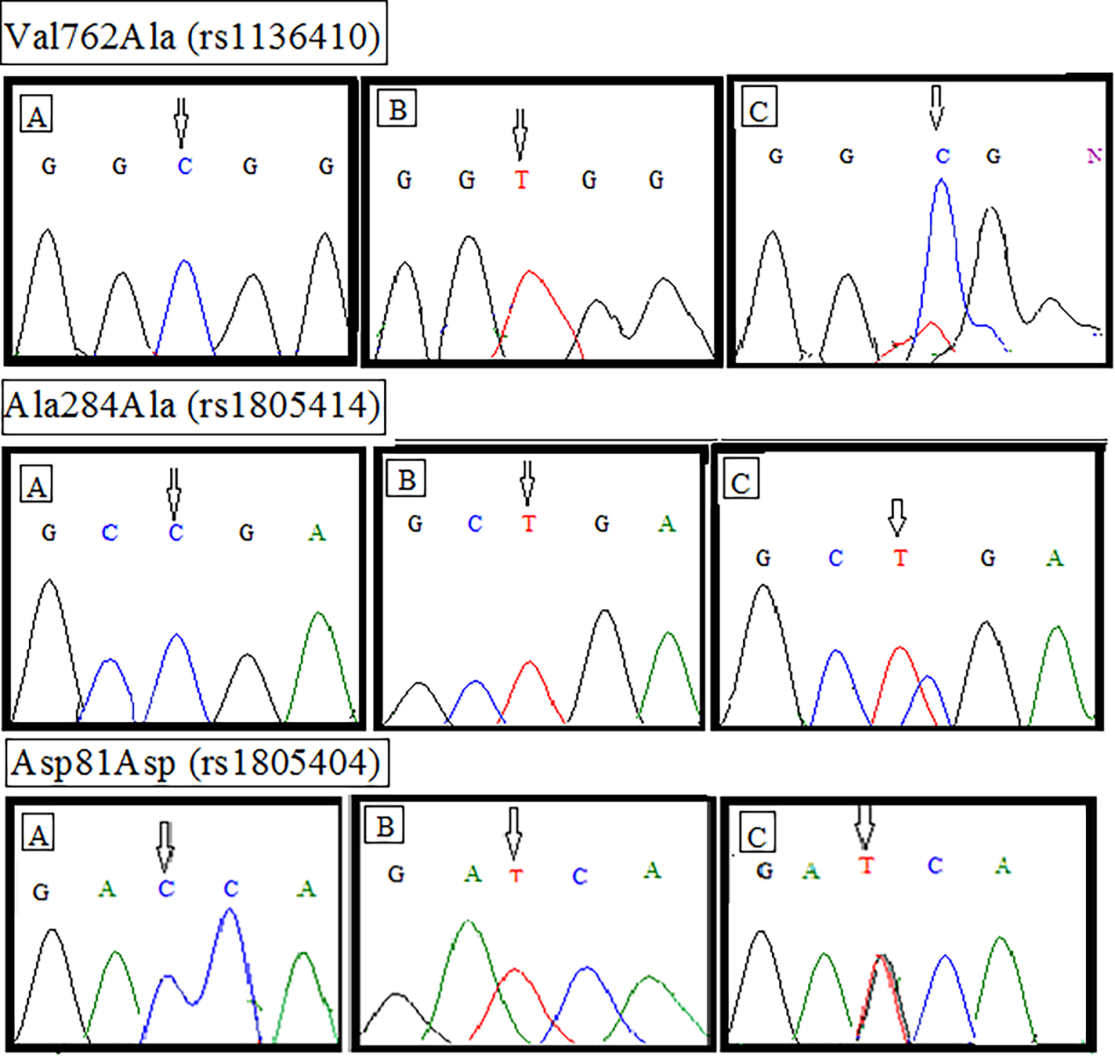
Electropherograms of selected polymorphism of *PARP1* gene in thyroid cancer patients and controls. Genotype pattern of first selected SNP, Val762Ala (rs3611410), (A) homozygous wild (B) homozygous mutant (C) heterozygous mutant. Genotype pattern of second selected SNP, Ala284Ala (rs1805414), (A) homozygous wild (B) homozygous mutant (C) heterozygous mutant. Genotype pattern of third selected SNP, Asp81Asp (rs1805404), (A) homozygous wild (B) homozygous mutant (C) heterozygous mutant.

Allele- specific polymerase chain reaction (ARMS-PCR) was performed for analyzing the changes in genetic make-up. Primers specific for the amplification of selected gene SNPs were designed using WASP (web-based allele specific primer designing tool) (Liu et al., 2012). Designed primers for each specific polymorphism are enlisted in S1 Table. Constituents of the PCR reaction were 50-100ng genomic DNA, 100μM of each primer and Solis BioDyne master mix whereas the total volume of the reaction was 10μl. Conditions for the thermal cycler reaction were: denaturation at 94°C for 30 secs, optimized annealing temperature for 45 secs, extension at 72°C for 1 min and final extension for 7 mins. Visualization of the PCR products was carried out through 2% agarose gel electrophoresis (100V, 300A for 45 mins). Results were anticipated based on the appearance of required bands for wild and mutant primers by UV trans illuminator (Gel Doc BioRad, USA). β-Actin was used as a positive control in each PCR reaction. Furthermore, the saples were sequenced to confirm the anticipated genotypes of each PCR product (wild, mutant and heterozygous) as shown in Fig 1.

### Statistical analyses

Chi square (χ^2^) test was used for comparing the demographical factors between cancer patients and controls for each SNP. Odd ratio (ORs) and 95% confidence intervals (CIs) were calculated according to the gender, age, smoking status and family history of cancer. Statistically significant P-value was <0.05. Multiplicative interaction effects of polymorphisms were estimated using adjusted logistic regression model for SNP-SNP interactions. Results were analyzed statistically by means of GraphPad prism software v 6.0.

From the genotypic data, haplotypes were generated. Examination of linkage disequilibrium (LD) and haplotype was performed using Haploview 4.2, which examines the data through expectation maximization (EM) algorithm. It gives the results of both haplotype and global data by estimating the haplotype frequency separately both in samples and controls. Odds ratios (ORs) and 95% CIs were assessed using unconditional logistic regression for each haplotype. Haplotype analysis was performed without accounting for the variables of the study because the haplotype analysis uses more degrees of freedom than single locus analysis. Moreover, statistical power decreases by adjusting the additional covariates. In haplotype analysis, each haplotype was evaluated separately versus all other haplotypes. In present study, genetic haplotype approach was used to analyze the influence of common cis ordered SNPs at the *PARP1* locus to thyroid cancer risk among Pakistani population. The order of variants in the inferred haplotypes was Val762Ala (rs3611410), Ala284Ala (rs1805414) and Asp81Asp (rs1805404), corresponding to the physical location of these variants in the *PARP1* gene. For examination, threshold frequency of haplotypes for inclusion was set at 1%. Power calculation was performed using Power Calculator for case-control Genetic Association Analysis (PGA), to assess the power value of the study. Input variables e.g. genetic mode of inheritance (co-dominant), disease allele frequency, marker allele frequency, control to case ratio and relative risk (RR) were used for power calculation at α = 0.05.

## Results

### Association of *PARP1* SNPs and risk of thyroid cancer

Genotype and allele frequencies of all the three selected SNPs of *PARP1* gene are listed in Table 2 and were calculated using Hardy-Weinberg equilibrium. For SNP rs3611410, frequency of minor allele homozygote (CC) was observed significantly higher in cancer patients (OR = 1.30; 95%CI 0.99–1.71; p = 0.05) compared to controls. For second SNP rs1805414, combined genotype (TC+CC) was observed significantly lower in thyroid cancer patients (OR = 0.43, 95% CI = 0.27–0.67; p = 0.003) compared to controls. Frequency of minor allele heterozygote (TC) was also observed significantly higher in healthy controls (OR = 0.80, 95% CI = 0.65–1.00; p = 0.05) compared to thyroid cancer patients. For SNP rs1805404, minor allele heterozygote (CT) was observed significantly higher in controls (OR = 0.63, 95% CI = 0.40–1.00; p = 0.05) compared to cancer patients as shown in Table 2.

**Table 2:** Allele and genotype frequencies of selected SNPs of *PARP1* gene in cases and controls.

Polymorphisms		Case, n(%)	Control, n(%)	OR (95% CI)	p^a^	Power^b^
*PARP1*						
rs3611410	TT	82 (17.98)	93 (23.25)	1.0 (Reference)		0.83
	TC	97 (21.27)	91 (22.75)	1.17 (0.85–1.62)	0.31	
	CC	276 (60.52)	216 (54.0)	1.30 (0.99–1.71)	**0.05**	
	CC+TC	373 (81.7)	307 (76.75)	1.36 (0.97–1.89)	0.06	
rs1805414	TT	70 (15.3)	29 (7.25)	1.0 (Reference)		0.39
	TC	268 (58.49)	291 (72.0)	0.80 (0.65–1.00)	**0.05**	
	CC	118 (25.8)	80 (20.0)	1.29 (0.94–1.77)	0.10	
	CC+TC	386 (84.6)	371 (92.7)	0.43 (0.27–0.67)	**0.003**	
rs1805404	CC	316 (69.2)	254 (63.5)	1.0 (Reference)		0.05
	CT	105 (23.2)	98 (24.5)	0.93 (0.69–1.27)	0.69	
	TT	35 (7.6)	48 (12.0)	0.63 (0.40–1.00)	**0.05**	
	TT+CT	140 (30.7)	146 (36.5)	0.77 (0.57–1.02)	0.07	

OR, odds ratio; CI, confidence interval; OR, CI and p^a^-value calculated by regression analysis.

Power^b^ Statistical power analysis using PGA1

Histological subtype analysis showed that minor allele homozygote CC (p = 0.02) of polymorphism rs1136410 was found significantly higher in papillary thyroid carcinoma compared to other subtypes of thyroid cancer. In case of polymorphism rs1805414, frequency of minor allele homozygote CC (p = 0.05) and minor allele heterozygote TC (p = 0.0001) was noted significantly higher in papillary thyroid carcinoma compared to follicular subtype, medullary and anaplastic subtype. In case of third polymorphism rs1805404, frequency of minor allele homozygote (TT) was observed significantly higher in papillary thyroid carcinoma (p = 0.008) and anaplastic carcinoma (p = 0.003) compared to other subtypes of thyroid carcinoma as shown in Table 3.

**Table 3:** Distribution of genotypes and odds ratios (OR) for different histological subtypes of thyroid carcinoma and controls.

Genotypes *PARP1*	Controls (n = 400)		Papillary carcinoma	*P- value		Follicular	*P- value		Medullary	*P- value		Anaplastic	*P- value
		n	(n = 351)		n	carcinoma		n	carcinoma		n	(n = 7)	
			OR (95% CI)			(n = 82)			(n = 16)			OR (95% CI)	
						OR (95% CI)			OR (95% CI)				
**rs1136410**													
TT	93	64	1.00(Ref)		15	1.00(Ref)		3	1.00(Ref)		4	1.00(Ref)	
TC	91	76	0.93(0.66–1.32)	0.71	17	0.88(0.49–1.59)	0.68	4	1.13(0.35–3.59)	0.83	1	0.56(0.06–4.76)	0.60
CC	216	217	1.37(1.03- 1.84)	**0.02**	48	1.20(0.74–1.94)	0.45	9	1.09(0.40–2.99)	0.85	2	0.340.06–1.77)	0.207
**rs1805414**													
TT	29	57	1.00(Ref)		13	1.00(Ref)		3	1.00(Ref)		1	1.00(Ref)	
TC	291	209	0.55(0.40–0.74)	**0.0001**	47	0.50(0.30–0.82)	**0.006**	9	0.48(0.17–1.32)	0.15	4	0.49 (0.11–2.26)	0.36
CC	80	91	1.40(0.99–1.97)	**0.05**	20	1.29(0.73–2.25)	0.37	4	1.33(0.41–4.24)	0.62	2	1.60(0.30–8.39)	0.57
**rs1805404**					** **		** **				** **		** **
CC	254	249	1.00(Ref)		55	1.00(Ref)		11	1.00(Ref)		2	1.00(Ref)	
CT	98	86	1.00(0.71–1.39)	0.99	18	0.86(0.48–1.53)	0.62	4	1.02(0.32–3.25)	0.96	1	0.51(0.06–4.31)	0.53
TT	48	22	0.49(0.28–0.83)	**0.008**	7	0.68(0.29–1.57)	0.37	01	0.48(0.06–3.78)	0.49	4	9.77(2.12–45.0)	**0.003**

OR, odds ratio; CI, confidence interval; OR, CI and *p-value calculated by regression analysis.

### Haplotype analysis results

Eight haplotypes were generated for three selected SNPs (rs1136410, rs1805414 and rs1805404) of *PARP1* gene among thyroid cases and controls. Haplotypes CTC has 25%, CTT has 46% and TCT has 28% reducing effect in thyroid cancer patients. TCC (OR = 1.33; 95%CI = 1.07–1.66; p = 0.01) and TTC (OR = 1.23; 95%CI = 0.99–1.52; p = 0.05) haplotypes were associated with increased risk of thyroid cancer as shown in Table 4. Global haplotype analysis showed that there was an overall significant difference in cases and controls (p = 0.001).

**Table 4:** Distribution of haplotype analysis in study cohort.

Haplotype	Cases	Controls	Chi^2^	Fisher’s P	Pearson’s P	OR (95% CI)
TTT	82.87 (0.091)	69.08 (0.086)	0.107	0.743	0.743	1.05 (0.75–1.47)
TTC	268.1 (0.294)	201.9 (0.252)	3.702	0.054	**0.054**	1.23 (0.99–1.52)
TCT	48.93 (0.054)	67.02 (0.084)	6.123	0.013	**0.013**	0.62 (0.42–0.90)
TCC	252.0 (0.276)	177.9(0.222)	6.588	0.010	**0.010**	1.33 (1.07–1.66)
CTT	23.88 (0.026)	37.71 (0.047)	5.399	0.020	**0.020**	0.54 (0.32–0.91)
CTC	134.1(0.147)	148.2 (0.185)	4.535	0.033	**0.033**	0.75 (0.58–0.97)
CCT	22.32 (0.024)	18.18 (0.023)	0.056	0.812	0.812	1.07 (0.57–2.01)
CCC	79.68 (0.087)	79.82 (0.100)	0.777	0.3780	0.378	0.86 (0.62–1.19)
Global			23.175	0.0016	0.0015	

Abbreviations: SNP, single nucleotide polymorphism; OR, odds ratio; CI, confidence interval; OR, CI and p-value calculated by regression analysis.

### Genotype-genotype interaction

After haplotype analysis, interaction between the selected SNP of *PARP1* gene was calculated by logistic regression model shown in Table 5. The analysis revealed that SNP 1 vs SNP 2 (Val762Ala vs Ala284Ala) and SNP2 vs SNP3 (Ala284Ala vs Asp81Asp) had a positive correlation with increased risk of thyroid cancer (OR = 1.099; 95% CI = 0.068–17.846; OR = 2.33; 95% CI = 0.142–37.366). However, SNP1 vs SNP3 (Val762Ala vs Asp81Asp) had a negative correlation with thyroid cancer risk (OR = 0.88; 95% CI = 0.532–1.486). All correlations were found statistically non-significant. These results were further confirmed by -2 log likelihood ratios of reduced model and no signification association between SNPs was observed (Table 6).

**Table 5:** Logistic regression model of SNP-SNP interactions and thyroid cancer risk.

Polymorphisms	B	S. E	Wald	Sig	OR	95% CI
rs3611410 vs rs1850414	0.095	1.42	0.004	0.947	1.099	0.068–17.846
rs3611410 vs rs1805404	-0.117	0.262	0.200	0.655	0.88	0.532–1.486
rs1850414 vs rs1805404	0.833	1.422	0.343	0.558	2.33	0.142–37.366

Abbreviations: SNP, single nucleotide polymorphism; OR, odds ratio; CI, confidence interval; OR, CI and p-value calculated by logistic regression analysis.

**Table 6:** Likelihood ratio analysis of SNP-SNP interaction in thyroid cancer patients.

Polymorphisms	-2 log likelihoods of reduced model	Chi-square	Sig
rs3611410 vs rs1850414	20.629	0.129	0.938
rs3611410 vs rs1805404	20.701	0.201	0.654
rs1850414 vs rs1805404	25.316	1.743	0.418

Abbreviations: SNP, single nucleotide polymorphism; significance level calculated by Likelihood ratio

Analysis

## Discussion

*PARP1* is an important DNA repair gene that binds with DNA and promote Poly (ADP-ribosylation) of many proteins. It plays a significant role in detecting the DNA damage and repair [26]. In this study, we investigated the association of three *PARP1* gene polymorphisms i-e Val762Ala (rs1136410), Ala284Ala (rs1805414) and Asp81Asp (rs1805404) with thyroid cancer susceptibility. We also explored whether the three polymorphisms were in linkage disequilibrium, and if any common haplotypes of these SNPs are found associated with thyroid carcinogenesis. At the end, we examined the combined effect of all three selected SNPs on thyroid cancer risk. Among these selected SNPs Val762Ala has been extensively studied. Substitution of an amino acid within carboxyl terminal of catalytic domain of the enzyme is responsible for *PARP1* Val762Ala variation. This variant can reduce the enzymatic activity by producing a steric modification in catalytic domain [27] and interaction with *XRCC1* is also limited [28]. These alterations can decrease the BER pathway capacity and hence increase the cancer susceptibility in *PARP1* Ala762 carriers. Therefore, genetic variants of *PARP1* that contribute to *PARP1* activity may be a risk factor for cancer development and progression. In thyroid cancer patients, significant higher minor homozygote (CC) frequency of Val762Ala was observed as compared to controls in present study. These results are consistent with previous studies that showed the rs1136410 as a risk factor for carcinomas of the brain, head and neck in Chinese population, esophagus, lung, breast, stomach, bladder, colorectal in European population, prostate and skin in USA [29–34]. However, contradictory results have also been obtained in glioma where rs1136410 plays a protective role [35]. In Caucasian populations, the *PARP1* Val762Ala gene variant minimizes the risk of development of some cancers [36–40], while in Chinese populations *PARP1* 762Ala gene variant increases the risk of cancer in numerous studies [41–43].

For second selected SNP of *PARP1* gene Ala284Ala (rs1805414), in cancer patients there was higher minor allele frequency than in controls and has significant effect on increasing the risk of thyroid cancer in current study. Previous studies have found a positive association between the Ala284Ala SNP and increased risk of breast cancer in Swedish population [44], Alzheimer’s disease in USA [16], Glioblastoma in German population [17], inversely associated with colorectal cancer in Chines population [18, 19]. A base substitution (T-allele) at Ala282Ala is not responsible for disrupting the protein function itself because, it does not alter the sequence of amino acid of that protein. So, the hypothesis of association of this variant with colorectal adenoma is because of the linkage disequilibrium with another variant in *PARP1* promoter is supported by our findings in current study [18]. Since regulation of *PARP1* gene is controlled by its promoter therefore, variation in this region at transcription binding sites may affect its expression [18]. Milani *et al*., (2007) [45] has also found that *PARP1* promoter region has functional regulatory polymorphisms and in cancerous cells, allelic imbalance influences the expression level of SNP rs1805414. In case of third polymorphism of *PARP1* gene Asp81Asp (rs1805404), frequency of minor allele homozygote was observed significantly higher in controls compared to thyroid cancer patients and showed inverse association with the said disease. Similar trend has earlier been reported by Jin *et al*. (2010) [46] where mutant allele of Asp81Asp has showed inverse association non-hedgehog lymphoma in males.

In second part of the study, the genotype frequency of three selected SNPs was compared with different histological subtypes of thyroid cancer and frequency of minor allele homozygote/heterozygote of Val762Ala (rs1136410), Ala284Ala (rs1805414) and Asp81Asp (rs1805404) was observed significantly higher in papillary thyroid carcinoma compared to other histological subtype of said disease. Similar trend for minor allele homozygote/heterozygote of DNA repair genes polymorphisms and increased incidence of papillary thyroid carcinoma compared to other histological subtype of thyroid cancer has been reported in different studies [47–50]. However, till now only one study has been reported with respect to *PARP1* gene polymorphism and thyroid cancer subtype and no significant change in crude/adjusted OR of Val762Ala polymorphism of *PARP1* gene, has been revealed in either papillary or follicular thyroid cancer subgroups [50].

In third part of the study, we successfully established haplotypes for the *PARP1* gene from the different combinations of the three SNPs. In global haplotype analysis, generally the significant difference was shown among patients and control individuals. This difference was because of the TTC and TCC haplotypes, which was more frequent in cases compared to controls, conferring increased effect against the development of thyroid cancer. Additionally, as far as we know, neither any study was carried out to analyze the combined effect in the form of haplotype analysis of Val762Ala (rs1136410), Ala284Ala (rs1805414) and Asp81Asp (rs1805404) SNPs of *PARP1* gene in thyroid cancer previously. When combining putative risk alleles in the form of haplotype, increase in risk of developing thyroid cancer was observed. Overall, this study findings propose a synergistic interaction among the susceptibility genotypes. It is suggested that increased effect observed, due to these susceptibility SNPs, is in agreement with poly allelic model, according to this model susceptibility is influenced by the combination of several alleles in a population, where each allele effect is responsible for a slight genotypic risk. Cancer growth and development is frequently affected by the SNPs with low penetrance. However, the combined effect of these variants is more effective than a single variant effect. [51]. The combined effect of *PARP1* polymorphism Val762Ala with other polymorphism have already be reported in different cancer such as lung cancer [43], noncardia gastric cancer [52], esophageal squamous cell carcinoma [36], skin cancer [53], breast cancer [32], colorectal cancer [39] and cancer [54].

To conclude, our findings indicate that selected SNPs of *PARP1* gene especially Val762Ala and some haplotypes of the said gene may play a role in increasing the thyroid carcinogenesis risk and its genetic susceptibility. However, the major coding SNPs, i.e., rs1805414 and rs1805404 polymorphism, of the *PARP1* showed inverse association with thyroid pathogenesis among Pakistani population. For confirming the contributions of single variation, gene-gene interactions and gene-environment interactions with thyroid cancer comprehensive studies should carried out in distinct ethnic groups because genetic polymorphisms differ between distinct groups.

## Supporting information

S1 Table Primers for *PARP1* gene SNPs with product size and annealing temperature. (DOCX)

S1 Annex Consent performa for the donors. (DOCX)
