# Supplementary material for: Interaction among susceptibility genotypes of PARP1 SNPs in thyroid carcinoma
Source: PLoS One. 2018 Sep 5;13(9):e0199007. doi: 10.1371/journal.pone.0199007 (PMC6124699; doi:10.1371/journal.pone.0199007)
Supplement: S1 Table — (DOCX) [file pone.0199007.s001.docx]

**S1 Table:** Primers for PARP1 gene SNPs with product size and annealing temperature

| Name of SNPs | Primer Sequence | Product  Size (bp) | Annealing  Temp. (^o^C) |
| --- | --- | --- | --- |
| rs1136410 |  | 236 | 60 |
| *WF | TTGCTCCTCCAGGCCAAGTC |  |  |
| **MF | TTGCTCCTCCAGGCCAAGTT |  |  |
| ***CR | CAGCTTTCCAGGAGATCCTA |  |  |
| rs1805414 |  | 153 | 54 |
| WF | GCAGATCTTGGACCGAGTAGAC |  |  |
| MF | GCAGATCTTGGACCGAGTAGAT |  |  |
| CR | GGTGTCTGTGTCTTGACCAT |  |  |
| rs1805404 |  | 213 | 56 |
| WF | TGAGCTTCGGTGGGATGTC |  |  |
| MF | TGAGCTTCGGTGGGATGTT |  |  |
| CR | GTGTTGCTGAAATAACATGG |  |  |

*Wild forward, ** Mutant Forward, *** Common Reverse
