# Supplementary material for: Interaction among susceptibility genotypes of PARP1 SNPs in thyroid carcinoma
Source: PLoS One. 2018 Sep 5;13(9):e0199007. doi: 10.1371/journal.pone.0199007 (PMC6124699; doi:10.1371/journal.pone.0199007)
Supplement: S1 Annex — (DOCX) [file pone.0199007.s002.docx]

COMSATS Institute of Information Technology

Department of Biosciences

Islamabad Campus

Consent Performa for the Donors

Blood: Tissue:

Status: Patient Normal

Hospital Ref No (PNR) Lab Ref no Date

Name W/o, D/o or S/o

Age Gender

Area of Cancer Year when disease started

Contact number Ethnic group/Caste

City

Marital Status Number of Children

Nature of Job Family history of cancer

Histological type of cancer:

Addiction (if any) like smoking, pan, chalia, naswar etc:

Remedies used (Iodine treatment, I-131 radiation, surgery, radiotherapy and thyroxin):

Treatment time

Any other complication like Goiter, nodules:

Any other Disease or surgery (other than thyroid):

Condition of thyroidism, Hypo or Hyperthyroidism:

Grade/Stage: TNM (tumor, node, metastasis):

Histopathological report:
